# Acute effects of combined and isolated caffeine and theanine supplementation on physical and cognitive performance in competitive athletes: a randomized, double-blind, placebo-controlled crossover study

**DOI:** 10.3389/fnut.2025.1751673

**Published:** 2026-01-26

**Authors:** Selin Yildirim Tuncer, Serhat Ozdenk, Ulas Can Yildirim, Dilara Erkan, Cengizhan Sari, Mehmet Can Gundem, Halit Sar, Busra Yilmaz, Izzet Karakulak, Firat Akca

**Affiliations:** 1 Department of Coaching Education, Faculty of Sport Sciences, Lokman Hekim University, Ankara, Türkiye; 2 Department of Sport Management, Faculty of Sport Sciences, Sinop University, Sinop, Türkiye; 3 Department of Coaching Education, Faculty of Sport Sciences, Sinop University, Sinop, Türkiye; 4 Department of Physical Education and Sports, Institute of Health Sciences, Ankara University, Ankara, Türkiye; 5 Department of Coaching Education, Faculty of Sport Sciences, Muş Alparslan University, Muş, Türkiye; 6 Department of Sport Management, Faculty of Sport Sciences, Sinop University, Sinop, Türkiye; 7 Department of Physical Education and Sport, Faculty of Sport Sciences, Sinop University, Sinop, Türkiye; 8 Department of Coaching Education, Faculty of Sport Sciences, Gazi University, Ankara, Türkiye; 9 Department of Sport Management, Faculty of Sport Sciences, Mardin Artuklu University, Mardin, Türkiye; 10 Department of Coaching Education, Faculty of Sport Sciences, Ankara University, Ankara, Türkiye

**Keywords:** caffeine, cognitive performance, co-supplementation, isometric strength, L-theanine

## Abstract

**Introduction:**

Modern athletic performance is driven not only by physical capacity but also by rapid decision-making, attentional control, and visuomotor coordination. Evidence regarding the acute effects of caffeine (CAF), L-theanine (TEA), and their combination remains inconsistent, particularly with respect to their combined influence on physical and cognitive performance in athletic populations. This study examined the acute effects of isolated and combined CAF and TEA supplementation on maximal strength, intermittent aerobic endurance, and eye–hand coordination in competitive athletes. It was hypothesized that the combined ingestion of CAF and TEA would differentially affect physical performance and eye-hand coordination outcomes compared with isolated CAF or TEA intake.

**Methods:**

Twenty trained athletes completed four randomized, double-blind, placebo-controlled crossover conditions: CAF (3 mg·kg^−^1), TEA (200 mg), CAF+TEA (COM), and placebo (CON), with ≥72 h washout. Outcomes included isometric leg, back, and handgrip strength; Yo-Yo Intermittent Endurance Test Level 1 performance; and visuomotor coordination (CogniFit^®^). Data were analyzed using repeated-measures ANOVA with Bonferroni corrections (α = 0.05).

**Results:**

Condition effects were found for leg strength (*p* = 0.004, ηp^2^ = 0.24) and back strength (*p* = 0.008, ηp^2^ = 0.19). In the COM condition, no additional or synergistic effect on maximal strength was observed. Additionally, no significant difference was found between the conditions in aerobic endurance, maximum isometric handgrip strength, and hand-eye coordination results. Caffeine ingestion did not differ from placebo for any strength outcome under the present conditions. Finally, acute ingestion of TEA was associated with reduced maximal isometric leg and back strength compared with CAF and CON.

**Discussion:**

Findings challenge the prevailing assumption of CAF + TEA synergy and underscore the need for task-specific interpretation of co-supplementation strategies. Future studies should evaluate dose–response interactions, habitual caffeine intake, sex-specific responses, and broader cognitive domains beyond visuomotor control. The randomized controlled trial was registered at ClinicalTrials.gov, under the registration number NCT07268573.

**Clinical Trial Registration:**

ClinicalTrials.gov, NCT07268573.

## 1 Introduction

The modern understanding of sport relies not only on high levels of physical capacity but also on rapid cognitive processing and decision-making skills. Today, in order for athletes to perform at a high level, the interaction of cognitive qualities such as attention, reaction time, strategic thinking and situational awareness is required, in addition to physical components such as strength, endurance, speed and agility. Particularly in team sports, instant tactical decisions and maintaining excellent visual-motor coordination are among the critical factors that determine the outcome of the match. Similarly, in individual sports, the athlete's internal attention regulation, stress control, and motor skill accuracy directly influence success. Therefore, the contemporary performance model requires the systematic development and support of cognitive performance in addition to physical preparation (1). These multi-dimensional performance requirements have encouraged research into ergogenic components that simultaneously support cognitive and physiological performance in recent years. Particularly in sports where cognitive functions are integrated with physical performance, nutritional supplements that enhance mental focus and decision-making speed are gaining increasing attention. Furthermore, regular caffeine consumption has been shown to be prevalent among physically active populations, who utilize it strategically to cope with physical demands and maintain alertness (2). In this context, the components caffeine (CAF) and L-theanine (TEA) have attracted attention due to their potential to create dual effects on the central nervous system and neuromuscular system, both individually and in combination (3, 4).

CAF (1,3,7-trimethylxanthine) is an adenosine receptor antagonist and has a stimulatory effect on the central nervous system. Acute CAF intake of approximately 3–9 mg·kg^−1^ body weight taken about 60 min before exercise stimulates the central nervous system, increasing fat oxidation, preserving glycogen, and delaying fatigue, thereby improving overall performance (5). Additionally, as a result of adenosine blockade, an increase in dopamine, norepinephrine, and acetylcholine levels is expected to reduce perceived exertion and increase alertness and motivation (6). These neurophysiological mechanisms contribute to performance enhancement in both aerobic and anaerobic exercise. Meta-analyses indicate that CAF consumption at doses of 3–6 mg·kg^−1^ increases aerobic endurance. It is emphasized that CAF intake significantly contributes to delayed fatigue and, consequently, prolonged exercise duration, particularly in protocols targeting aerobic performance (5, 7, 8). Similarly, comprehensive analyses of strength and power parameters indicate that CAF supplementation produces statistically significant increases in lower and upper body strength and muscle power (6, 9, 10). In addition to its physical benefits, CAF reliably enhances cognitive performance, including critical attributes such as reaction time and alertness, for athletes working under high pressure and time-critical conditions (5). Although caffeine's stimulant effects can enhance performance, its use alone may not always be ideal, as it can cause anxiety, palpitations, restlessness, and excessive arousal in some athletes (11). Therefore, it has become important to investigate neuromodulators that can balance the cognitive and physiological effects of CAF.

In this context, TEA (γ-glutamyl-ethylamide), an amino acid naturally found in green tea leaves, has become a noteworthy ergogenic component due to its unique neurocognitive properties. TEA can provide relaxation and stress reduction without creating a sedative effect on the central nervous system (12). Neurochemically, it has been reported to increase levels of serotonin, dopamine, and gamma-aminobutyric acid (GABA) in the brain; it also enhances alpha wave activity, creating a calm yet alert mental state (13, 14). These effects may positively influence cognitive performance components such as extended attention span, increased concentration, and maintenance of emotional balance under stress (3, 4). In the context of exercise, it is suggested that TEA may alleviate physiological stress responses by balancing the levels of stress hormones such as cortisol and adrenaline during demanding tasks (15, 16) and thus contribute to maintaining endurance under fatigue (12, 17). However, some studies indicate that acute TEA supplementation (≈50–400 mg), does not have a significant effect on cognitive or physical performance, or that the effects are limited to specific tasks (18, 19). These findings warrant a cautious assessment of claims regarding TEA's independent ergogenic effects on athletic performance. However, studies on the direct effects of TEA on exercise performance parameters (strength, endurance, etc.) are still limited, and the results are not entirely consistent (20–22). Therefore, further experimental research is needed to elucidate the neurophysiological mechanisms and dose-response relationships of TEA on sports performance.

The combination of CAF and TEA has gained importance in recent years due to its potential synergistic effect in the field of cognitive performance. The balancing of CAF's stimulant properties with TEA's calming effect creates an aroused yet controlled state of attention. One study reported that a combination of 97 mg TEA + 40 mg CAF increased attention, accuracy, and subjective alertness (3). In another study using a similar combination, improvements in reaction time and accuracy were reported (4). Furthermore, it was reported that this combination produced stronger effects than the individual components in task switching and sustained attention tasks (23). These findings suggest that CAF and TEA together can optimize cognitive performance and may reduce the potential side effects of CAF (e.g., tremor, restlessness). The potential synergistic effect of CAF and TEA is explained by the opposing yet complementary effects of the two compounds on the autonomic nervous system and neurotransmitter release. CAF increases central arousal and cognitive activation by enhancing dopaminergic and noradrenergic activity via adenosine receptor antagonism (6). In contrast, TEA creates a calming effect by increasing serotonin, dopamine, and GABA levels, thereby supporting parasympathetic tone (14, 24). This dual-action neuromodulation is thought to support more stable arousal, lower mental noise, faster response, and more efficient motor output, particularly during exercise under stress. Therefore, the synergistic effect of the combination may stem not from a single biological mechanism, but from the interaction of multiple processes such as arousal regulation, neurotransmitter modulation, and the synchronization of attention networks. Furthermore, the combined effects of CAF + TEA on physical performance have not been sufficiently clarified. Most previous studies have focused primarily on short-term cognitive outcomes such as attention, reaction time, and task switching (14, 23). In contrast, research examining physical performance variables such as strength or aerobic capacity remains scarce. Although CAF has strong findings in terms of strength and aerobic endurance, TEA has rarely been examined alone or in combination with CAF in relation to such physical performance outcomes (21, 22). For example, in elite curling athletes, TEA intake alone and in combination with CAF led to improvements in shot accuracy and decision-making indicators (22). In elite wrestlers, the acute ingestion CAF + TEA (3 mg·kg^−1^ each), compared to placebo, led to increases in power/strength and sport-specific endurance outputs, as well as cognitive response (21). Although these studies show that the combination of CAF and TEA improves physical and cognitive performance compared to placebo, it is also mentioned that this combination produced ineffective results in some parameters. In the study by Razazan et al. (21), no significant difference was observed in jump and throw performance when the CAF + TEA condition was compared to the CAF condition alone. Due to the contradictory nature of these data, further research is needed. In this context, the theoretical complementarity of the physiological and neurocognitive effects of CAF and TEA suggests that these two components, when used together, may create a potential synergy in both central and peripheral performance outcomes. However, to the authors' knowledge, there are almost no controlled experimental studies that directly test this possibility and simultaneously assess strength, aerobic endurance, and cognitive performance under the same protocol. Furthermore, previous studies examining cognitive performance have mostly focused on reaction time and attention, but no research examining fine motor control, such as hand-eye coordination, has been found. The variability in the reported effects of caffeine and caffeine-containing mixtures may be partly due to differences in habitual caffeine intake and withdrawal protocols. These differences are not controlled for uniformly across studies.

In view of the limited and conflicting findings to date, the aim of this study is to investigate the effects of CAF and TEA supplementation, both individually and in combination, on acute strength, aerobic endurance, and cognitive performance in competitive athletes. Accordingly, the hypothesis was proposed that taking CAF and TEA together would provide superior improvements in both physical (strength and aerobic endurance) and cognitive performance compared to taking either substance separately.

## 2 Materials and methods

### 2.1 Study design

This study employed a randomized, double-blind, placebo-controlled, crossover design to examine the effects of caffeine (CAF), theanine (TEA), their combination (COM), and a placebo (CON) on physical and cognitive performance in young, healthy, competitive athletes. Each participant completed all four experimental conditions in a randomized order, with a washout period of at least 72 h between sessions to minimize potential carryover effects. The randomization sequence was generated by an independent researcher using a computer-based randomization tool (25). To ensure familiarity and minimize learning effects, all participants completed a familiarization session 1 week prior to the experimental trials. Physical and cognitive performance were assessed across multiple primary outcomes, as pre-specified in the trial registration, including maximal strength, intermittent aerobic endurance, and eye-hand coordination. All procedures were conducted in accordance with the Declaration of Helsinki. The study protocol was approved by the Sinop University Human Research's Ethics Committee (Approval No: 2025/337), and all participants provided written informed consent prior to participation.

### 2.2 Participants

Twelve male and eight female national athletes from both team (basketball, handball, and soccer) and individual (badminton, tennis) sports were recruited for the study. During the first laboratory visit, anthropometric and body composition assessments were performed to characterize the participant sample. Standing height and body mass were measured to the nearest 0.1 cm and 0.1 kg using a calibrated stadiometer and digital scale (Seca, Hamburg, Germany). Body composition was determined via multi-frequency bioelectrical impedance analysis (InBody 770; InBody Co., Seoul, South Korea). All measurements were conducted in the morning to minimize metabolic effects, with participants wearing light clothing and having abstained from food and caffeine for at least 3 h prior to testing to standardize gastrointestinal conditions.

Inclusion criteria were: (i) active participation in individual or team sports at a competitive level, (ii) age between 18 and 30 years, and (iii) absence of any known medical or metabolic disorder. Exclusion criteria included: (i) history of cardiovascular, neurological, or musculoskeletal disorders within the past 12 months, (ii) known intolerance or hypersensitivity to caffeine or theanine, (iii) allergy to mannitol or other excipients used in capsule formulation, (iv) current use of prescription medications, and (v) physician-directed restriction of caffeine or theanine consumption.

The required sample size was estimated using an *a priori* power analysis (G^*^Power 3.1.9.7; Düsseldorf, Germany) for a repeated-measures ANOVA with four conditions (CAF, TEA, CAF + TEA, CON), assuming a moderate effect size (*f* = 0.25), α = 0.05, and statistical power (1 – β) = 0.80. The analysis indicated a minimum required sample of 16 participants. To account for potential attrition or missing data, 20 athletes were recruited; all completed the study protocol.

### 2.3 Supplementation protocol

Capsules were ingested 60 min prior to testing to allow sufficient time for peak plasma caffeine concentrations (26). The supplementation conditions were structured as follows: caffeine (CAF, 3 mg·kg^−1^ anhydrous caffeine), theanine (TEA, 200 mg L-theanine), combined caffeine and theanine (COM, 3 mg·kg^−1^ caffeine + 200 mg theanine), and placebo (CON, mannitol). Caffeine capsules contained anhydrous caffeine powder (Oxford brand pure caffeine powder; ISO 14001 certified; 98.5% purity; The Oxford Vitality Health Company Ltd., London, UK), and theanine capsules contained pure L-theanine powder Ocean (L-Theanine, 200 mg; Orzax Pharmaceuticals, Istanbul, Turkey; active ingredient manufactured in Germany). Placebo capsules contained mannitol, a non-nutritive sugar alcohol with no known ergogenic or ergolytic properties (27). To achieve the target caffeine dose of 3 mg·kg^−1^ while maintaining a constant capsule number across all experimental conditions, capsules were individually prepared for each participant. In the CAF condition, the total calculated caffeine dose was adjusted according to body mass and evenly distributed across five capsules. In the COM condition, the same body-mass–adjusted caffeine dose (3 mg·kg^−1^) was combined with a fixed dose of 200 mg L-theanine, with the caffeine and theanine contents distributed across the same five capsules. In the TEA condition, capsules contained 200 mg L-theanine without caffeine, whereas in the placebo condition, capsules were filled exclusively with inert excipients. This approach ensured that each participant ingested a total of five capsules in all conditions, with identical capsule number, total mass, and external appearance, thereby preserving blinding and reproducibility across trials. Neither participants nor investigators could distinguish between conditions based on capsule characteristics.

Participants were instructed to refrain from alcohol consumption and strenuous exercise for 24 h before each trial and to abstain from any dietary supplements throughout the study period. They maintained 24-h dietary records for the day preceding each test and a weekly log of their habitual caffeine intake. To standardize pre-trial nutritional status, participants replicated their recorded dietary intake and hydration patterns before each visit. To preserve ecological validity and reduce potential caffeine withdrawal effects, participants were permitted and encouraged to maintain their usual daily caffeine consumption during the study period, as recommended by Tallis et al. (28) and Pickering and Kiely (29).

### 2.4 Data collection tools

All sessions were conducted under standardized computer laboratory and indoor sports hall conditions. The eye-hand coordination assessment was conducted in a quiet, temperature-controlled laboratory using a desktop computer system. Ambient conditions were maintained at approximately 22 ± 1 °C and 50 ± 5% relative humidity. All other performance tests (strength and aerobic endurance) were carried out in an indoor sports hall under comparable conditions (temperature 21–23 °C, relative humidity 45%−55%). Environmental parameters were monitored at the start of each session using a calibrated digital room thermo-hygrometer. Lighting, background noise, and testing times were standardized across all trials to ensure intra-individual consistency.

Testing sessions were held in the morning at 09:00 a.m. to minimize the potential effects of circadian variation and diurnal performance fluctuations. The sequence of tests was standardized as follows: eye-hand coordination, maximal strength, and aerobic endurance. Adequate rest intervals were provided between tests to prevent fatigue and carryover effects. The following measurements were collected during each experimental condition.

### 2.5 Strength test

Maximal isometric strength was assessed using a Lafayette digital dynamometer (Takei Scientific Instruments Co. Ltd, Tokyo, Japan) to measure handgrip, back, and leg strength. The device was checked for calibration prior to each testing session according to the manufacturer's recommendations.

Participants performed three maximal voluntary contractions for each test, with at least 60 s of rest between attempts to minimize fatigue. Handgrip strength was assessed with participants seated, shoulders adducted, elbows flexed at 90°, and the dynamometer handle adjusted to individual hand size. Back strength was measured in a standing position with the participant pulling upward on a handle attached to the dynamometer's base platform while maintaining a neutral spine. Leg strength was evaluated in a similar posture, with participants exerting maximal upward force through a chain-linked platform while keeping their knees slightly flexed.

The mean of the three trials for each test was recorded as the participant's strength score, expressed in kilogram-force (kgf) as displayed by the device. All strength assessments were performed in accordance with the standardized procedures for isometric testing recommended by the American College of Sports Medicine (30).

### 2.6 Aerobic endurance

Aerobic endurance was assessed using the Yo-Yo Intermittent Endurance Test Level 1 (Yo-Yo IET-1), which evaluates participants' ability to perform repeated bouts of exercise with brief recovery periods. The protocol followed the standardized procedures described by Bangsbo (31). Testing was performed on a flat, non-slip indoor surface with two parallel lines marked 20 m apart and a recovery line placed 5 m behind the starting line. Prior to testing, participants completed a standardized 5-min dynamic warm-up consisting of jogging and mobility drills (31).

Participants ran back and forth between the 20-m lines in time with audio cues from the official Yo-Yo IET Level 1 recording, with running speed progressively increasing throughout the test. After each 2 × 20 m shuttle, a 5-s active recovery period was provided, during which participants jogged to and from the recovery line. The test was terminated when a participant failed to reach the 20-m line on two consecutive occasions, and the total distance covered (m) was recorded to the nearest meter as the performance outcome.

### 2.7 Eye-hand coordination

Eye-Hand Coordination Test (Multidirectional and Unpredictable Direction) was used to evaluate participants' ability to perform accurate, continuous manual motor actions based on dynamic visual processing. The test was administered in a quiet, temperature-controlled laboratory (22 ± 1 °C; relative humidity 50 ± 5%) using CogniFit's proprietary digital platform (CogniFit Inc., New York, USA) on a desktop computer.

They were instructed to remain seated comfortably, maintain a neutral wrist position, and use their dominant hand for all trials. During testing, participants tracked a small moving circle (“ball”) that followed an unpredictable multidirectional trajectory. The task required maintaining a cursor (crosshair) within the moving circle as precisely as possible. Visual feedback was provided continuously: green when on-target, orange when near the boundary, and red when the cursor left the target area. Each assessment included two initial learning segments (7 s slow + 7 s fast) followed by twelve testing segments alternating between slow (≈5 cm·s^−1^) and fast (≈10 cm·s^−1^) movement speeds, each lasting either 2,333 ms (short) or 7,000 ms (long), for a total duration of approximately 5–7 min including instructions. Prior to the main test, participants completed a brief familiarization session that included standardized on-screen practice trials lasting approximately 1 min to minimize learning effects. Optional 10–15 s rest intervals were allowed between trials to avoid fatigue.

*The following performance metrics were recorded automatically by the CogniFit software:*

*Accuracy (%):* Percentage of time the cursor remained inside the circle (0–100; higher = better).

*Accuracy - Fast Speed (%):* accuracy during fast segments (≥10 cm·s^−1^).

*Accuracy - Slow Speed (%):* accuracy during slow segments ( ≤ 5 cm·s^−1^).

*Accuracy - Long Segments (%):* accuracy during 7,000 ms segments (sustained tracking).

*Accuracy - Short Segments (%):* accuracy during 2,333 ms segments (brief tracking).

*Distance from Circle Center (pixels):* mean pixel distance between cursor and target center (0–1,200; lower = better precision).

Performance scores were automatically normalized against CogniFit's age- and sex-matched normative database (percentile 0–100), following the developer's validity index ranges and completion-time criteria (54–58 s valid window). All assessments adhered to the manufacturer's standardized task protocol and validity thresholds (32).

### 2.8 Statistical analysis

The normality of the data set was initially evaluated using the Shapiro-Wilk test. Subsequently, the assumption of sphericity was examined through Mauchly's test, with the Greenhouse-Geisser correction applied in cases where sphericity was violated. Differences in strength, aerobic endurance, and eye–hand coordination outcomes across the four supplementation conditions (CAF, TEA, COM, and CON) were analyzed using a one-way repeated-measures analysis of variance (ANOVA). When significant main effects were observed, Bonferroni-adjusted pairwise comparisons were conducted to identify specific between-condition differences. The magnitude of effects was quantified using partial eta squared (ηp^2^), interpreted as small (0.10–0.24), moderate (0.25–0.39), or large (≥0.40). Data were presented as mean ± SD. Between-condition mean differences and pairwise effect sizes (Cohen's *d* for repeated measures) are reported with their 95% confidence intervals (CI95%) to indicate estimate precision and facilitate interpretation beyond *p*-values alone. Effect sizes were interpreted as trivial (< 0.20), small (0.20–0.49), moderate (0.50–0.79), or large (≥0.80) (33). In addition to the primary analyses, potential period and order effects were examined by incorporating session number and treatment order into exploratory analyses. No significant period, order, or treatment-by-period effects were observed (*p* > 0.05), suggesting that the washout period (≥72 h) was sufficient to minimize carryover effects. All statistical analyses were performed using IBM SPSS Statistics version 30.0 (IBM Corp., Armonk, NY, USA) and statistical significance was accepted at α = 0.05 (two-tailed). Graphical visualizations were generated with GraphPad Prism version 10.0 (GraphPad Software, San Diego, CA, USA).

## 3 Results

**Figure 1:**
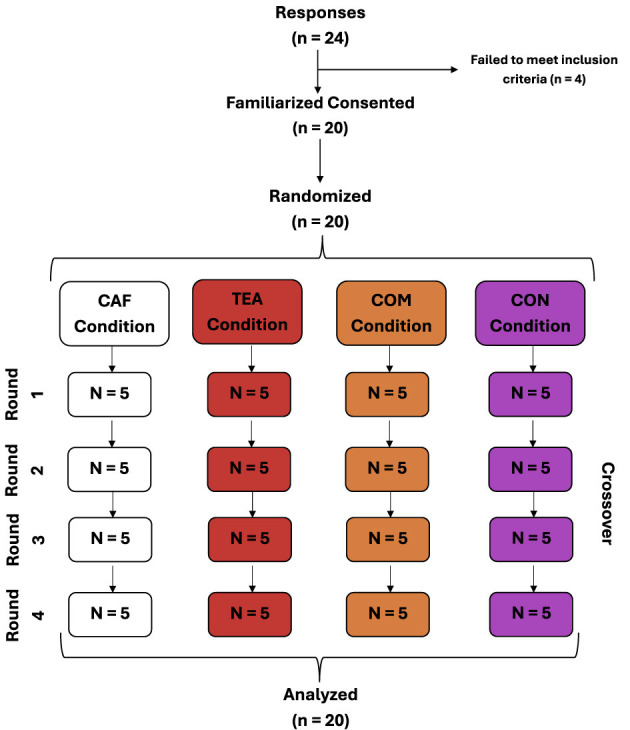
Flowchart.

Figure 1 illustrates the participant flow and crossover allocation throughout the study. Twenty-four athletes initially responded to the study invitation, of whom four were excluded for not meeting the inclusion criteria. Twenty athletes were familiarized with the procedures, provided informed consent, and were randomized. All randomized participants completed a four-condition, four-round crossover design, receiving CAF, TEA, COM, and CON in a randomized order. No dropouts occurred, and all participants completed all experimental conditions and were included in the final analyses. Participant characteristics were as follows: age 19.9 ± 2.4 years, height 167.3 ± 7.6 cm, body mass 63.5 ± 10.2 kg, lean body mass 27.5 ± 6.7 kg, body fat percentage 22.6 ± 8.2%, body mass index (BMI) 22.6 ± 2.3 kg·m^−2^, and habitual caffeine intake 318.8 ± 72.2 mg·day^−1^.

**Figure 2:**
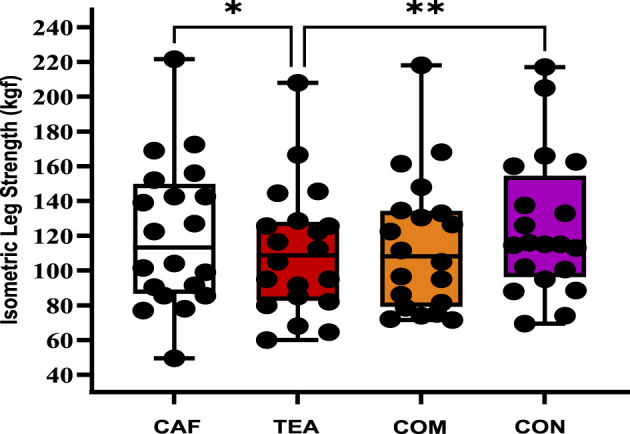
Isometric leg strength performance (mean ± SD) across different supplementation conditions. Individual data points are shown as black dots, with bars representing mean ± SD; *n* = 20 per condition. CAF, caffeine; TEA, theanine; COM, combined; CON, control; *p < 0.05, **p < 0.01 (data analyzed using repeated-measures ANOVA with Bonferroni *post-hoc* test).

Figure 2 shows the effects of different supplementation conditions on leg strength performance. A significant difference of condition was observed (*p* = 0.004, ηp^2^ = 0.24), indicating a small-to-moderate effect size according to predefined thresholds. Bonferroni-adjusted pairwise comparisons showed that leg strength was significantly higher in the CAF condition (120.3 ± 41.6 kgf) compared to TEA [111.1 ± 36.79 kgf; *p* = 0.022; 95% CI (1.04, 17.46); *d* = 0.24]. Similarly, leg strength in the CON condition (124.9 ± 39.96 kgf) was greater than in TEA [111.1 ± 36.79 kgf; *p* = 0.03; 95% CI (−23.70, −3.95); *d* = 0.35]. No other pairwise comparisons reached statistical significance under the present experimental conditions.

**Figure 3:**
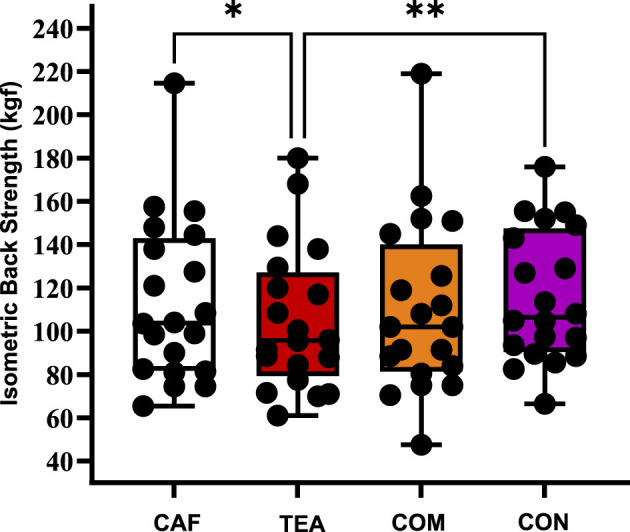
Isometric Back strength performance across different supplementation conditions. Individual data points are shown as black dots, with bars representing mean ± SD; *n* = 20 per condition. CAF, caffeine; TEA, theanine, COM, combined, CON, control, *p < 0.05, **p < 0.01 (data analyzed using repeated-measures ANOVA with Bonferroni *post-hoc* test).

Figure 3 shows the effects of different supplementation conditions on back strength performance. A significant difference of condition was observed (*p* = 0.008, ηp^2^ = 0.19), indicating a small-to-moderate effect size. Similarly, back strength in the CON condition (115.90 ± 30.41 kgf) was greater than in TEA [105.02 ± 33.03 kgf; *p* = 0.008; 95% CI (−19.43, −2.32); *d* = 0.31]. No other pairwise differences were statistically significant under the present experimental conditions.

**Figure 4:**
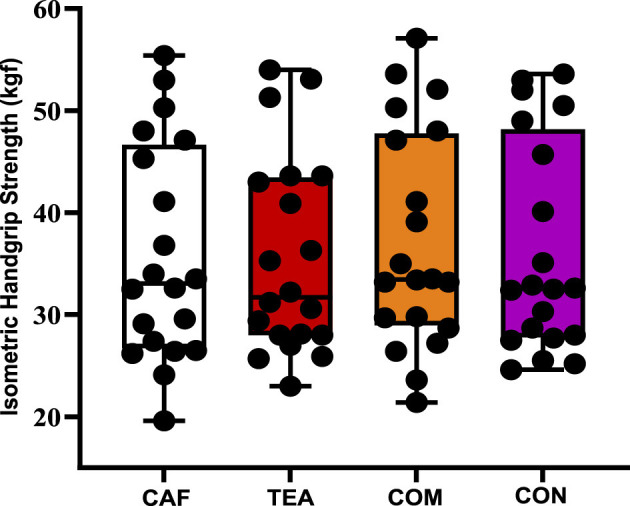
Isometric Hand grip strength performance across different supplementation conditions. Individual data points are shown as black dots, with bars representing mean ± SD; *n* = 20 per condition. CAF, caffeine; TEA, theanine; COM, combined; CON, control.

Figure 4 shows the effects of different supplementation conditions on handgrip strength. Mean values were 35.9 ± 10.6 kgf (CAF), 35.5 ± 9.7 kgf (TEA), 37.2 ± 10.7 kgf (COM), and 36.3 ± 10.4 kgf (CON). Statistical analysis revealed no detectable effects under the present experimental conditions (*p* = 0.105, ηp^2^ = 0.105), indicating a small effect size.

**Figure 5:**
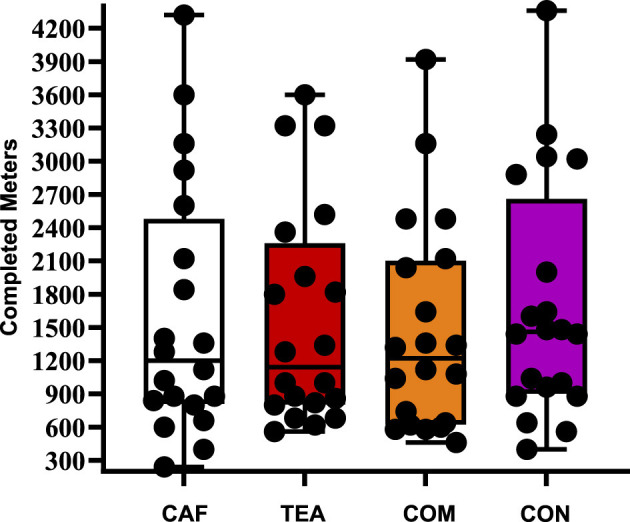
Yo-Yo intermittent endurance test level 1 performance across different supplementation conditions. Individual data points are shown as black dots, with bars representing mean ± SD; *n* = 20 per condition. CAF, caffeine; TEA, theanine; COM, combined; CON, control.

Figure 5 presents the mean distances achieved in the Yo-Yo Intermittent Endurance Test Level 1 across supplementation conditions. Mean performance values were 1,602 ± 1,149 m (CAF), 1,561 ± 985 m (TEA), 1,466 ± 959 m (COM), and 1,699 ± 1,067 m (CON). Statistical analysis revealed no detectable effects under the present experimental conditions (*p* = 0.223, ηp^2^ = 0.076), indicating a small effect size.

Table 1 summarizes the mean values, standard deviations, mean differences relative to placebo, and corresponding effect sizes for isometric strength and intermittent endurance outcomes across supplementation conditions. Compared with placebo, caffeine and the combined condition did not produce statistically significant differences in leg strength, back strength, handgrip strength, or Yo-Yo IET1 performance (*p* > 0.05). In contrast, L-theanine was associated with lower leg and back strength values compared with placebo, indicating a negative effect on maximal isometric force production. Effect sizes for all comparisons, suggesting limited practical significance under the present experimental conditions.

**Table 1:** Acute effects of caffeine, L-theanine, and their combination on strength and intermittent endurance outcomes compared with placebo.

**Parameters**	***N* = 20**	**Mean**	**Standard deviation**	**Mean difference (Δ) CAF compared to CON Mean (95%CI) Effect size (*d*)**	**Mean difference (Δ) TEA compared to CON Mean (95%CI) Effect size (*d*)**	**Mean difference (Δ) COM compared to CON Mean (95%CI) Effect size (*d*)**
Leg strength (kgf)	CAF	120.32	41.61	−4.57 (−16.71 to 7.56) *d* = −0.11	−13.82 (−23.70 to −3.95) *d* = −0.35	−10.45 (−24.05 to 3.15) *d* = −0.26
	TEA	111.08	36.79			
	COM	114.45	39.05			
	CON	124.90	39.96			
Back strength (kgf)	CAF	113.47	37.34	−2.42 (−12.83 to 7.98) *d* = −0.07	−10.87 (−19.43 to −2.32) *d* = −0.307	−5.80 (−17.04 to 5.44) *d* = −0.164
	TEA	105.02	33.03			
	COM	110.10	39.98			
	CON	115.90	30.42			
Handgrip strength (kgf)	CAF	35.92	10.60	−0.42 (−2.22 to 1.38) *d* = −0.04	−0.84 (−2.70 to 1.03) *d* = −0.08	0.83 (−0.90 to 2.56) *d* = 0.08
	TEA	35.51	9.72			
	COM	37.17	10.74			
	CON	36.34	10.36			
YOYO IET1 (meters)	CAF	1,602	1,149.30	−97 (−323.68 to 129.68) *d* = −0.09	−138 (−444.92 to 168.92) *d* = −0.13	−233 (−644.04 to 178.04) *d* = −0.22
	TEA	1,561	985.89			
	COM	1,466	959.48			
	CON	1,699	1,067.32			

CAF, caffeine; TEA, theanine; COM, combined; CON, control.

Data were analyzed using one-way repeated-measures ANOVA across four supplementation conditions (CAF, TEA, COM, and CON). When a significant main effect was detected, Bonferroni-adjusted pairwise comparisons were performed. Mean differences (Δ) are presented relative to the placebo condition (CON). Effect sizes are reported as Cohen's d for repeated measures with corresponding 95% confidence intervals.

Table 2 summarizes the mean scores and variability measures for eye-hand coordination parameters across supplementation conditions. No significant effects of condition were found for any of the coordination outcomes (*p* > 0.05).

**Table 2:** Eye–hand coordination performance variables (mean ± SD) across supplementation conditions.

**Parameters**	**Condition *N* = 20**	**M ±SD**	***f-*Value**	***p-*Value**	**ηp^2^**
EHC-A	CAF	97.329 ± 2.199	0.704	0.546	0.036
	TEA	96.976 ± 2.154			
	COM	96.636 ± 2.571			
	CON	97.310 ± 1.733			
EHC-FS	CAF	96.279 ± 3.403	1.453	0.243	0.071
	TEA	95.954 ± 3.017			
	COM	94.839 ± 4.320			
	CON	96.681 ± 2.171			
EHC-SS	CAF	98.388 ± 1.954	0.540	0.620	0.028
	TEA	97.961 ± 2.100			
	COM	98.332 ± 1.593			
	CON	97.853 ± 1.886			
EHC-LSD	CAF	97.463 ± 2.095	0.421	0.718	0.022
	TEA	97.065 ± 2.184			
	COM	96.915 ± 2.788			
	CON	97.367 ± 1.414			
EHC-DFBC	CAF	9.592 ± 1.873	1.258	0.298	0.062
	TEA	9.643 ± 1.959			
	COM	9.848 ± 2.183			
	CON	9.146 ± 1.799			

CAF, caffeine; TEA, theanine; COM, combined; CON, control; EHC, eye hand coordination; A, accuracy; FS, fast speed; SS, slow speed; LSD, long segments duration; DFBC, distance from the ball center.

Data were analyzed using one-way repeated-measures ANOVA across the four supplementation conditions (CAF, TEA, COM, and CON). F-values, *p*-values, and partial eta squared (ηp^2^) are reported as measures of statistical significance and effect size.

Accuracy (EHC-A) values were similar among conditions (*p* = 0.546, ηp^2^ = 0.036). Fast-speed accuracy (EHC-FS) also showed no significant differences (*p* = 0.243, ηp^2^ = 0.071). Similarly, no significant between-condition effects were observed for slow-speed accuracy (EHC-SS; *p* = 0.620, ηp^2^ = 0.028), long-segment duration accuracy (EHC-LSD; *p* = 0.718, ηp^2^ = 0.022), or the average distance from the ball center (EHC-DFBC; *p* = 0.298, ηp^2^ = 0.062). All observed effect sizes were classified as small.

## 4 Discussion

This study examined the effects of CAF, TEA, their COM, and CON on acute physical and cognitive performance in young competitive athletes. The results indicate that the performance effects of these supplements were task-specific and limited in magnitude. Notably, caffeine did not differ from placebo in terms of any outcome. Additionally, while TEA use resulted in a statistically significant decrease in maximum isometric leg and back strength, no significant effect was observed for any condition in parameters such as hand grip strength, intermittent endurance, or hand-eye coordination.

When the study findings were examined, it was observed that although the combined (CAF + TEA) condition had numerically higher values compared to L-theanine alone, it did not show a significant difference from placebo in terms of maximal leg/back isometric strength performance. The observed differences between COM and TEA conditions may reflect the negative impact of L-theanine alone on performance, rather than an interaction attributable to caffeine. Overall, these findings indicate no additive or synergistic benefit of co-ingestion under the present conditions. In a study of healthy adults, both regular/irregular caffeine consumers, under controlled caffeine abstinence, 50 mg of L-theanine administered with 75 mg of caffeine was reported to antagonize the effects of caffeine alone, but not provide any significant benefit (34).

The strength test findings showed comparable response patterns for both leg and back isometric strength. It was observed that CAF (3 mg·kg^−1^) administration did not provide a detectable effect in isometric leg strength compared to the CON group (*p* > 0.05). The literature contains studies reporting that caffeine has ergogenic effects on isometric strength under specific experimental conditions. Behrens et al. (35), in a study on healthy adults who were active in their leisure time under controlled caffeine abstinence, showed that a higher dose of caffeine (8 mg·kg^−1^) significantly increased the maximum isometric torque of knee extensors. Similarly, in healthy adults who regularly consumed moderate amounts of caffeine and underwent resistance training with controlled caffeine abstinence prior to testing, a significant increase in isometric knee extensor performance was reported following caffeine intake of 6 mg·kg^−1^ compared to placebo (36). However, these studies were generally conducted using isokinetic systems (e.g., Biodex) that test isolated muscle groups. The dynamometer test used in the current study is a standing strength measurement, described in the literature as multi-joint and complex, similar to movements such as leg thrusts. Such functional movements require synchronized activation of the quadriceps and hamstrings, gluteus, and core stabilizer muscle groups (37, 38). Consistent with this notion, Yildirim and Akçay (39) reported that caffeine intake increased quadriceps muscle strength but had no significant effect on hamstring muscle strength. The researchers explained this difference by suggesting that caffeine has a greater potential to increase motor unit recruitment via the central nervous system in muscle groups that exhibit lower neural activation during maximal voluntary contractions. In this context, the leg strength test used in the present study may have reduced the sensitivity to detecting minor caffeine-related effects because it requires the simultaneous activation of multiple muscle groups and postural stabilization.

In the current study, 3 mg·kg^−1^ of caffeine did not detectable effects maximal isometric back strength compared to the control group. Consistent with current findings, a study in healthy, recreationally active adult men under controlled caffeine abstinence reported that, despite a higher caffeine dose (6 mg·kg^−1^), caffeine did not significantly alter peak isometric force production of lumbar extensors during the Biering-Sørensen test compared to placebo (40). The Biering–Sørensen test is similar to the dynamometry test used in the current study in that it primarily requires isometric contraction of the back extensor muscles (41). Other studies have similarly reported no significant improvement in strength performance in the Isometric Mid-Thigh Pull (IMTP) test following acute supplementation with 3 mg·kg^−1^ of caffeine in trained individuals (42, 43). The test setup used to measure back strength in the current study is structurally similar to the IMTP protocol (44). Accordingly, the current findings regarding the effects of caffeine on maximal isometric back strength performance are consistent with previous literature.

The literature suggests that the effects of caffeine on maximal isometric strength are highly dependent on muscle group, test modality, and task characteristics (45). Therefore, the sensitivity for detecting caffeine-related effects may be reduced in isometric tests measuring back and leg strength, which require multiple muscle activation of the posterior chain and involve synergistic muscle control. Higher doses or alternative testing protocols may be needed to detect caffeine-related effects in tasks involving the simultaneous activation of large muscle groups; however, this is still speculative.

Acute 200 mg L-theanine intake did not enhance performance in either test context (leg/back strength), and in fact, it potentially reduced it compared to placebo. Therefore, the findings suggest that acute L-theanine intake may have an ergolytic-like effect under the current test conditions, rather than leading to overall performance impairment. Research on the effects of L-theanine on acute physical performance is relatively recent. A review of the literature reveals that various performance metrics have been evaluated, such as sport-specific skills, anaerobic power (RAST, vertical jump), balance and fatigue index, and handgrip strength. However, no study to date has specifically examined maximal isometric strength with the inclusion of both leg and back tests. While improvements in some physical performance outcomes were observed in experimental conditions using only L-theanine, TEA does not provide consistent ergogenic effects across all metrics. In a study by Jebelli et al. (20) acute theanine ingestion (200 mg) significantly improved balance and minimal anaerobic power, while causing a significant decrease in mean anaerobic power. In another study, 3 mg·kg^−1^ L-theanine intake did not produce any significant differences compared to placebo in any physical performance metric such as wall squat time, vertical jump, medicine ball throw, handgrip strength, or sport-specific tests (21). In a study evaluating sport-specific throwing and cognitive skills following controlled caffeine deprivation and acute L-theanine supplementation (6 mg·kg^−1^) in elite curling athletes, no consistent performance improvement was observed (22).

At the molecular level, L-theanine has been shown to modulate central nervous system activity, which may partly account for the observed reductions in maximal isometric strength; however, this interpretation remains speculative. Structurally similar to glutamate, L-theanine binds to glutamate receptors such as α-Amino-3-hydroxy-5-methyl-4-isoxazolepropionic acid (AMPA), kainite, and N-methyl-d-aspartate (NMDA), acting as a weak antagonist of these receptors (46, 47). In addition, L-theanine has been shown to increase gamma-aminobutyric acid (GABA) levels and GABA receptor density. As an inhibitory neurotransmitter, GABA induces a state of neurological calmness and relaxation (48). This dual effect, which involves suppressing the glutamate system and supporting the GABA system, may allow L-theanine to induce a calming yet focused neurological state. While these neurochemical effects may be beneficial for tasks requiring attentional control or fine motor coordination, they may be less favorable for tasks that rely on high cortical excitability and maximal motor unit recruitment, such as maximal isometric strength tests (49). However, these mechanistic interpretations remain speculative, as no neurophysiological measures (e.g., EMG, EEG, or perceived exertion) were collected in the present study. In this context, the findings suggest that the effects of L-theanine may be context-specific and do not provide a general ergogenic benefit on physical performance.

In the present study, when isometric hand grip strength findings were examined, neither caffeine L-theanine combination nor the L-theanine alone showed a statistically significant difference compared to placebo (*p* > 0.05). When comparing TEA and CAF, L-theanine decreased performance, although not statistically significantly. These results are consistent with previous findings in elite wrestlers (21).

3 mg·kg^−1^ caffeine supplementation did not significantly increase isometric handgrip strength in our study (*p* = 0.105). This result is consistent with the small but significant effect size (Cohen's *d* = 0.17) reported in Grgic's (50) meta-analysis. The meta-analysis combined the findings of 16 studies and reported that caffeine had an ergogenic effect compared to placebo, which was statistically significant but quite small in terms of effect size (Cohen's *d* = 0.17). The ηp^2^ = 0.105 obtained in the current study also indicates a small effect size. However, the lack of statistical significance suggests that any potential effect of this magnitude is likely limited and should be interpreted cautiously under the current circumstances. In conclusion, although the observed effect size was small, it is unlikely that the reinforcement protocol applied in our study has a significant practical effect on isometric hand grip strength.

According to the Yo-Yo Intermittent Endurance Test findings, no supplementation condition had a significant effect on performance. The lack of a significant effect of 3 mg·kg^−1^ caffeine on Yo-Yo Intermittent Endurance Test performance compared with placebo (*p* = 0.223) is consistent with the literature highlighting substantial inter-individual variability in caffeine's ergogenic effects, which may be partly explained by caffeine habituation (51). A recent experimental study shows that regular caffeine consumption may reduce acute ergogenic responses to moderate caffeine doses (52). In the current study, the sample group was allowed to continue their habitual caffeine intake, which may have reduced responsiveness to the acute 3 mg·kg^−1^ dose. Acute administration of 200 mg L-theanine also did not reach statistical significance on the Yo-Yo Intermittent Endurance Test (*p* = 0.223). This finding is consistent with the results of Jebelli et al. (20), who reported that 200 mg L-theanine did not significantly alter the fatigue index measured by the Repetitive Anaerobic Sprint Test (RAST) in trained Wushu athletes (*p* = 0.190). Despite methodological differences in endurance protocols, both studies show that acute L-theanine supplementation did not significantly improve resistance to fatigue or endurance performance during high-intensity interval exercise. When the findings of the present study are examined, the high standard deviations observed in Yo-Yo IET1 scores were noteworthy (e.g., 1,602 ± 1,149 m). This may partly reflect the heterogeneity within the present sample of athletes. Such heterogeneity may have contributed to increased inter-individual variability and potentially reduced the sensitivity to detect small condition-specific effects, particularly in the combined (CAF + TEA) condition. The literature indicates that Yo-Yo intermittent test performance can vary substantially depending on sport type and competitive level (53).

Cognitive assessment revealed that acute administration of CAF, TEA, or COM did not significantly affect hand-eye coordination compared to the control group. ηp^2^ values ranged from 0.022 to 0.071, indicating no practical significance. This neutral effect in the COM group partially aligns with the findings of Camfield et al. (17), who reported modest improvements in attention outcomes following combined administration of CAF and TEA, but showed no significant effect on reaction time, intersensory attention, or mood. While some studies have shown COM-related improvements in tasks involving executive functions, such as the Stroop test, the current discrepancy may stem from task-specific neurophysiological demands. The EHC test primarily assesses sustained perceptual tracking, sensorimotor integration, and fine motor accuracy. While the CAF-TEA combination promotes rapid attention shifting, L-theanine's ability to reverse caffeine-induced vasoconstriction (34) may inhibit arousal necessary for sustained visual-motor performance. Therefore, the cognitive benefits observed in tasks like the Stroop test may not translate into improved hand-eye coordination.

Despite the findings contributing to the literature, the current study has several limitations. (1) While our study sample size (*n* = 20) was sufficient to detect moderate effects according to *a priori* power analysis (*f* = 0.25, power = 0.80), the effects of supplements such as caffeine are frequently reported as “small” in the literature. Therefore, small effect sizes or non-significant results, especially for measures such as handgrip strength or the Yo-Yo test, eye-hand coordination should be re-evaluated with a larger sample. (2) Our participant group was heterogeneous, including both male (*n* = 12) and female (*n* = 8) athletes. While this allows for inference to the general population, the sample size is insufficient to analyze potential gender-based differences in supplement responses. (3) Methodologically, participants were allowed to continue their habitual caffeine consumption. This choice limited the ability to control for caffeine tolerance and the effects of withdrawal. Although habitual caffeine intake was quantified (318.8 ± 72.2 mg·day^−1^), the present study was not designed to formally evaluate moderation effects of habitual caffeine consumption on acute responses. (4) Genetic factors that could influence individual differences in caffeine response (e.g., CYP1A2 or ADORA2A polymorphisms) were not controlled for. (5) Finally, measurement of cognitive performance was limited to eye-hand coordination, a complex visual-motor skill. The lack of attention or executive function tasks such as the Stroop test or measures reflecting central mechanisms such as the rating of perceived exertion (RPE) limited our ability to fully assess the potential cognitive effects of supplements.

Given these limitations, future research should be conducted with larger, sex-balanced samples. The antagonistic or synergistic effects of different doses and ratios of caffeine and L-theanine, particularly on maximal strength, should be clarified. Furthermore, abstinence protocols should be implemented to isolate the effects of caffeine habituation and genetic polymorphisms, and the relationship between different cognitive tasks (e.g., simple reaction time and executive functions) and physical performance should be examined.

## 5 Conclusion

In conclusion, this study examined the effects of acute L-theanine, caffeine or their combination on physical and cognitive performance in young competitive athletes. One of the main findings of our study is that acute ingestion of 200 mg L-theanine was associated with lower maximal isometric leg and back strength compared to placebo. This effect, observed under current experimental conditions, can be explained by possible neuroinhibitory mechanisms, but direct measurements to confirm these pathways have not been taken.

Another noteworthy finding was that the caffeine and L-theanine combination did not demonstrate any superiority over placebo. Additionally, taking caffeine and L-theanine together did not provide any additional or synergistic benefits. These findings emphasize that the interaction between L-theanine and caffeine is highly task-specific, and that the stress-reducing or cognitive benefits of L-theanine may not be directly generalizable to all performance domains, such as maximal strength.

From a practical standpoint, the observed effects appear to be task-specific, and the key signal is the potential ergolytic effect of acute L-theanine intake on maximal isometric strength. Accordingly, caution may be warranted when considering L-theanine supplementation before tasks requiring maximal force production.

## Data Availability

The raw data supporting the conclusions of this article will be made available by the authors, without undue reservation.
